# A Clever Trick Exposed

**Published:** 1885-06

**Authors:** 


### A CLEVER TRICK EXPOSED.

An impudent nostrum vender of Rochester, N. Y., cut out of our April number a portion of the editorial on General Grant's case, and adding thereto a puff for his so-called "kidney-cure," succeeded in getting the whole published as reading matter in a number of the leading dailies throughout the United States, representing the same to be entirely from our journal. Very many persons who do not see the *Homœopathist* have thus been led to believe that we lent ourselves to such quackery. We never indorsed this or any other nostrum, and the editorial columns of the *Homœopathist* are not for sale. It was a clever trick to steal the cloak of respectability to cover his nephritic nostrum, and the audacious individual probably supposed that we would supinely submit to such misrepresentation; but he has already discovered that he woke up the wrong customer. Immediately upon the appearance of this fraudulent notice we telegraphed to its author that we should demand exemplary legal damages for his unwarranted use of our name. The daily papers in New York gladly rectified the matter as far as they were able when their attention was called to it; but it's a lively truth that can catch up to a lie that has twenty-four hours' leeway.

We will be very grateful to any of our readers who, having seen this advertisement (printed as reading matter) in their local press, will cause a correction to be inserted. It is impossible for the editor of the *Homœopathist* to know where or when this matter may crop up, and he will be greatly obliged to any friend who will aid him in sitting down heavily on this brazen knave.—*From the American Homœopathist for May.*

